# Supplementary material for: A UMLS-based spell checker for natural language processing in vaccine safety
Source: BMC Med Inform Decis Mak. 2007 Feb 12;7:3. doi: 10.1186/1472-6947-7-3 (PMC1805499; doi:10.1186/1472-6947-7-3)
Supplement: Additional file 13 — RAP application directory. Sets up directory for RAP (RDF application for PHP) source code [file 1472-6947-7-3-S13.gz › rap/doc/phpdoc/__filesource/fsource_model__modelBlanknode.php.html]

xml version="1.0" encoding="iso-8859-1"?


File Source for Blanknode.php


# Source for file Blanknode.php

Documentation is available at Blanknode.php

```
1. <?php

3. // ----------------------------------------------------------------------------------
4. // Class: BlankNode
5. // ----------------------------------------------------------------------------------


11. /**
12. * An RDF blank node.
13. * In model theory, blank nodes are considered to be drawn from some set of
14. * 'anonymous' entities which have no label but are unique to the graph.
15. * For serialization they are labeled with a URI or a _:X identifier.
16. *
17. * <BR><BR>History:<UL>
18. * <LI>07-27-2003                : BlankNode can now receive both MemModel and DbModel as parameter
19. * <LI>02-21-2003                : getLabel() added.</LI>
20. * <LI>09-10-2002                : First version of this class.</LI>
21. * </UL>
22. *
23. * @version  V0.9.1
24. * @authors Chris Bizer <chris@bizer.de>,
25. *           Radoslaw Oldakowski <radol@gmx.de>
26. *
27. * @package model
28. * @todo nothing
29. * @access    public
30. *
31. */
32. class BlankNode extends Resource {

34. /**
35. * Constructor
36. * You can supply a label or You supply a model and a unique ID is gernerated
37. *
38. * @param    mixed    $namespace_or_uri_or_model
39. * @param     string $localName
40. * @access    public
41. * @todo     nothing
42. */
43. function BlankNode($namespace_or_uri_or_model , $localName = NULL) {

45. if (is_a($namespace_or_uri_or_model, 'Model')) {
46. // generate identifier
47. $id = $namespace_or_uri_or_model->getUniqueResourceURI(BNODE_PREFIX);

49. $this->uri = $id;

51. } else {
52. // set identifier
53. if ($localName == NULL) {
54. $this->uri = $namespace_or_uri_or_model;
55. } else {
56. $this->uri = $namespace_or_uri_or_model . $localName;
57. }
58. }
59. }

61. /**
62. * Returns the ID of the blank node.
63. * @return     string
64. * @access    public
65. */
66. function getID() {
67. return $this->uri;
68. }

70. /**
71. * Returns the ID of the blank node.
72. * @return     string
73. * @access    public
74. */
75. function getLabel() {
76. return $this->uri;
77. }

79. /**
80. * Dumps bNode.
81. *
82. * @access    public
83. * @return    string
84. */
85. function toString() {

87. return 'bNode("' . $this->uri . '")';
88. }

90. /**
91. * Checks if two blank nodes are equal.
92. * Two blank nodes are equal, if they have the same temporary ID
93. *
94. * @access    public
95. * @param        object    resource $that
96. * @return    boolean
97. */
98. function equals ($that) {

100. if ($this == $that) {
101. return true;
102. }
103. if (($that == NULL) or !(is_a($that, 'BlankNode'))) {
104. return false;
105. }

107. if ($this->getURI() == $that->getURI()) {
108. return true;
109. }

111. return false;
112. }

114. } // end: BlankNode


117. ?>
```

Documentation generated on Thu, 7 Jul 2005 13:41:08 +0200 by phpDocumentor 1.3.0RC3
